# The neurobiological basis of sex differences in learned fear and its inhibition

**DOI:** 10.1111/ejn.14602

**Published:** 2019-11-07

**Authors:** Harriet L. L. Day, Carl W. Stevenson

**Affiliations:** ^1^ School of Biosciences University of Nottingham Loughborough UK; ^2^ Present address: RenaSci Ltd BioCity, Pennyfoot Street Nottingham NG1 1GF UK

**Keywords:** discrimination, extinction, fear conditioning, female, generalization, male

## Abstract

Learning that certain cues or environments predict threat enhances survival by promoting appropriate fear and the resulting defensive responses. Adapting to changing stimulus contingencies by learning that such cues no longer predict threat, or distinguishing between these threat‐related and other innocuous stimuli, also enhances survival by limiting fear responding in an appropriate manner to conserve resources. Importantly, a failure to inhibit fear in response to harmless stimuli is a feature of certain anxiety and trauma‐related disorders, which are also associated with dysfunction of the neural circuitry underlying learned fear and its inhibition. Interestingly, these disorders are up to twice as common in women, compared to men. Despite this striking sex difference in disease prevalence, the neurobiological factors involved remain poorly understood. This is due in part to the majority of relevant preclinical studies having neglected to include female subjects alongside males, which has greatly hindered progress in this field. However, more recent studies have begun to redress this imbalance and emerging evidence indicates that there are significant sex differences in the inhibition of learned fear and associated neural circuit function. This paper provides a narrative review on sex differences in learned fear and its inhibition through extinction and discrimination, along with the key gonadal hormone and brain mechanisms involved. Understanding the endocrine and neural basis of sex differences in learned fear inhibition may lead to novel insights on the neurobiological mechanisms underlying the enhanced vulnerability to develop anxiety‐related disorders that are observed in women.

## Graphical abstract

We review studies on sex differences in learned fear inhibition. Overall, females show less fear extinction and discrimination than males, involving sex differences in the underlying brain areas. Estrogen regulates fear extinction and discrimination in females. Understanding the neurobiological basis of sex differences in learned fear inhibition may shed light on why women are more prone to anxiety, which is linked to deficits in learned fear inhibition and relevant neural circuit function.

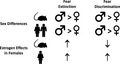

**Abbreviations**
ACC: anterior cingulate cortex
BDNF: brain‐derived neurotrophic factor
BLA: basolateral amygdala
CeA: central amygdala
COMT: catechol‐O‐methyltransferase
CS: conditioned stimulus
DH: dorsal hippocampus
ERα: estrogen receptor α
ERβ: estrogen receptor β
IL: infralimbic cortex
La: lateral amygdala
LTP: long‐term potentiation
mPFC: medial prefrontal cortex
pERK: phosphorylated extracellular signal‐regulated kinase
PFC: prefrontal cortex
PL: prelimbic cortex
PTSD: post‐traumatic stress disorder
SNP: single nucleotide polymorphism
TrkB: tropomyosin‐related kinase B
US: unconditioned stimulus
vmPFC: ventromedial prefrontal cortex

## 1 INTRODUCTION

Anxiety and trauma‐related disorders can be serious forms of psychiatric disease associated with a huge socioeconomic burden, given their significant lifetime prevalence and inadequate treatment options with currently available psychological therapies and medications. The etiology of anxiety‐related disorders is complex and involves interactions between various genetic and environmental factors (Craske et al., 2017). Epidemiological evidence indicates a clear role for biological sex in determining the vulnerability to develop anxiety‐related disorders, such that their prevalence is up to twice as high in women compared to men (Kornfield, Hantsoo, & Epperson, 2018; Lebron‐Milad & Milad, 2012; McLean, Asnaani, Litz, & Hofmann, 2011; Perrin et al., 2014). Yet even though this risk is widely acknowledged to be markedly greater in women than in men, there is still relatively little known about the neurobiological mechanisms involved.

**Figure 1:**
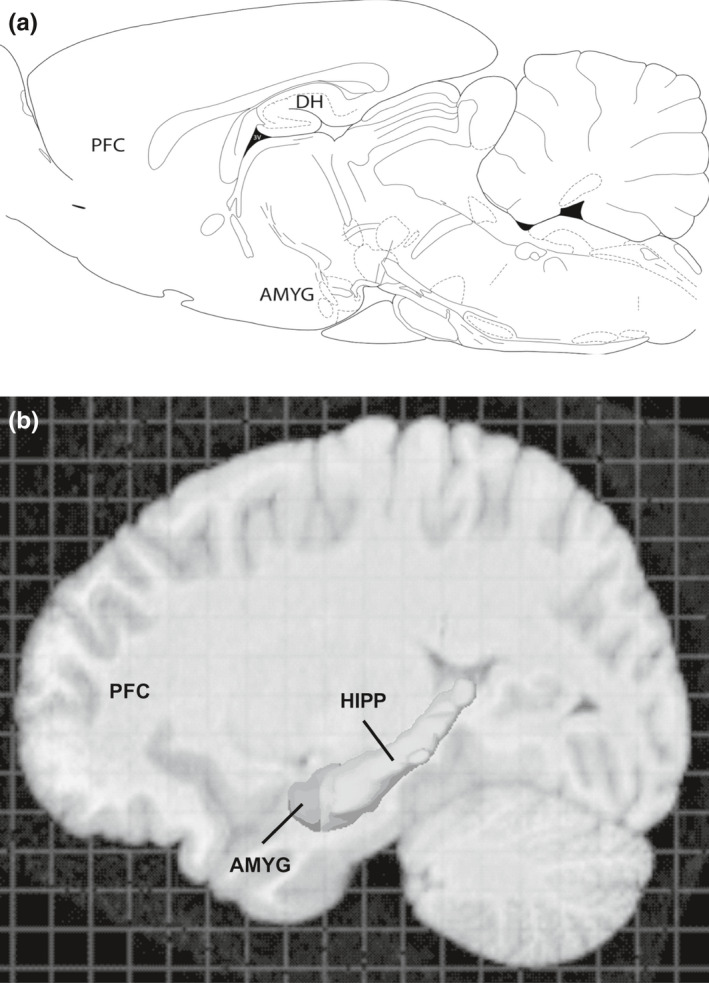
Brain areas involved in learned fear and its inhibition in (a) rats (adapted from Paxinos & Watson, 2007) and (b) humans (adapted from Allen Human Brain Atlas; Ball, Gilbert, & Overly, 2012). AMYG: amygdala; DH; dorsal hippocampus; HIPP: hippocampus; PFC: prefrontal cortex

Certain anxiety‐related disorders are characterized by abnormally persistent emotional memories of fear‐related stimuli, and impaired inhibition of learned fear is thought to be an endophenotype of such diseases, including post‐traumatic stress disorder (PTSD) (Jovanovic, Kazama, Bachevalier, & Davis, 2012; Singewald & Holmes, 2019; Zuj, Palmer, Lommen, & Felmingham, 2016). Investigating the neural circuit basis of learned fear and its inhibition from a translational perspective has been possible using broadly similar experimental paradigms in rodent and human studies. This research has identified several homologous brain areas (e.g., amygdala, hippocampus, prefrontal cortex (PFC); Figure 1) across species that play crucial roles in various Pavlovian fear learning and memory processes, including the inhibition of learned fear. Importantly, this neural circuitry is also implicated in the cognitive and emotional regulation deficits that are key features of anxiety and PTSD (Asok, Kandel, & Rayman, 2019a; Dunsmoor & Paz, 2015; Sevenster, Visser, & D'Hooge, 2018; Tovote, Fadok, & Lüthi, 2015). Preclinical studies determining the neural mechanisms underpinning learned fear inhibition have therefore been pivotal for gaining a better understanding of the pathophysiology underlying these disorders. However, until recently, most of these animal studies have only examined the inhibition of learned fear and its neural circuit basis in males (Lebron‐Milad & Milad, 2012). Investigating the neurobiological underpinnings of sex differences in learned fear inhibition may thus shed light on the mechanisms that mediate the greatly increased prevalence of anxiety‐related disorders that are observed in women in comparison with men.

In this narrative review, we begin by providing a brief overview of the various factors involved in sex differences in anxiety‐related disorders before reviewing the literature on sex differences in Pavlovian fear learning and memory. We then focus on the growing body of evidence from rodent and human studies indicating important sex differences in two types of learned fear inhibition: extinction and discrimination. Our coverage of both fear extinction and fear discrimination distinguishes this review from other recent reviews on the topic (Garcia, Walker, & Zoellner, 2018; Jasnow, Lynch, Gilman, & Riccio, 2017; Li & Graham, 2017; Ramikie & Ressler, 2018; Velasco, Florido, Milad, & Andero, 2019). Emerging evidence indicates that these sex differences in learned fear inhibition involve gonadal hormone signaling and are accompanied by altered function of the underlying neural circuitry. We then conclude with future directions to take this important area of research forward. While there are also key sex differences in the effects of stress on the encoding and inhibition of learned fear (Maeng & Milad, 2015; Merz & Wolf, 2017), this topic is beyond the scope of the present review.

## 2 OVERVIEW OF SEX DIFFERENCES IN ANXIETY‐RELATED DISORDERS

It is now well established that there are marked sex differences in certain fear‐ and stress‐related psychiatric diseases, such as anxiety‐related disorders. For example, women are up to 60% more likely to suffer from an anxiety disorder and up to twice as likely to suffer from PTSD, compared to men. Women are more likely to be diagnosed with co‐morbid anxiety, eating and mood disorders, and experience a greater burden with illness than men (Lebron‐Milad & Milad, 2012; McLean et al., 2011). Women are also more likely to report greater severity and persistence of PTSD symptoms, with these sex differences remaining when the exposure frequency to traumatic events is equivalent between women and men (Breslau, 2002; Seedat, Stein, & Carey, 2005). The reasons for these discrepancies in disease course between the sexes remain poorly understood but are thought to be multifactorial in nature. These factors include hormonal status, stress reactivity, temperament, cognition, environmental effects and societal influences (Catuzzi & Beck, 2014; McLean & Anderson, 2009). Different trauma types and co‐morbid disorders are associated with different levels of PTSD risk. At the same time, there are differences in the types of trauma to which women and men are more likely to be exposed and in the likelihood of having co‐morbidities that are linked to increased PTSD risk. For example, sexual abuse, which is more commonly and frequently experienced by women than men, is associated with a higher PTSD risk compared to other types of trauma. Such factors might contribute to sex differences in PTSD prevalence, although they are unlikely to account for them fully (Kornfield et al., 2018; Perrin et al., 2014).

Recent research has identified genetic factors involved in moderating anxiety and PTSD risk in a sex‐dependent manner. Several studies have examined the link between gene variants of catechol‐O‐methyltransferase (COMT), an enzyme important for metabolizing the neurotransmitter dopamine in certain brain areas (e.g., PFC), and anxiety‐related traits or disorders in men and women (Harrison & Tunbridge, 2008). Studies have shown an association between COMT genotype and symptoms or traits related to panic and anxiety that was specific to women (Domschke et al., 2004; Olsson et al., 2005; Stein, Fallin, Schork, & Gelernter, 2005), while other studies have reported a link between COMT genotype and anxiety endophenotypes or panic disorder in males only (Konishi, Tanii, Otowa, Sasaki, Motomura, et al., 2014; Konishi, Tanii, Otowa, Sasaki, Tochigi, et al., 2014; Lee & Prescott, 2014). However, some studies have also found an association that was independent of sex, or found no such association in either sex (Hoth et al., 2006; Howe at al., 2016; Wray et al., 2008). Differences in the specific gene variants involved, population demographics (e.g., ethnicity), and the diagnostic or trait criteria used between the studies might help to explain these discrepancies. More recent studies have discovered a role for a variant of the gene encoding the PAC1 receptor in influencing PTSD risk and symptoms specifically in women. The PAC1 receptor mediates signaling by pituitary adenylate cyclase‐activating polypeptide, an important regulator of cellular responses to stress (Hammack & May, 2015). A single nucleotide polymorphism (SNP) in the PAC1 receptor has been linked to PTSD diagnosis and symptom severity selectively in women (Ressler et al., 2011; Almli et al., 2013; Lind et al., 2017; but see also Chang et al., 2012). PAC1 receptor genotype has also been associated with emotional numbing symptoms in traumatized mothers (Wang et al., 2013). Interestingly, this SNP is located in an estrogen response element and a recent study has shown interactions between PAC1 receptor genotype, circulating estradiol levels and symptom severity in women with PTSD (Mercer et al., 2016).

## 3 SEX DIFFERENCES IN FEAR LEARNING AND MEMORY ENCODING

A feature of phobias and PTSD in particular is the abnormal persistence of emotional memory for fear‐related cues or contexts. Preclinical studies examining various (typically Pavlovian) fear learning and memory processes have proved useful as animal models of relevance to this feature of such anxiety‐related disorders. During Pavlovian fear conditioning, a neutral conditioned stimulus (CS), such as a discrete cue (e.g., sound) or a context (e.g., testing chamber), is associated with an aversive unconditioned stimulus (US; e.g., mild electric shock). Following conditioning, the association between the CS and US consolidates into long‐term memory. Later presentation of the conditioned cue or re‐exposure to the conditioned context initially results in the expression of conditioned fear responding, which has typically been inferred by quantifying freezing behavior elicited by the CS (Tovote et al., 2015).

Rodent studies have investigated sex differences in Pavlovian fear conditioning and memory retrieval. Many studies have shown reduced contextual fear conditioning in females, compared to males, as indicated by decreased freezing during the later retrieval of contextual fear memory (Maren, Oca, & Fanselow, 1994; Gupta, Sen, Diepenhorst, Rudick, & Maren, 2001; Wiltgen, Sanders, Behne, & Fanselow, 2001; Gresack, Schafe, Orr, & Frick, 2009; Chang et al., 2009; Ribeiro et al., 2010; Barker & Galea, 2010; Bethancourt, Vásquez, & Britton, 2011; Daviu, Andero, Armario, & Nadal, 2014; Ujjainwala, Courtney, Wojnowski, Rhodes, & Christian, 2019; Clark, Drummond, Hoyer, & Jacobson, 2019; but see Blume et al., 2017). Sex differences in nociception during conditioning are unlikely to explain this finding given the evidence for enhanced shock sensitivity in females (Dalla & Shors, 2009). Studies have found that females show more locomotor activity than males (Aguilar et al., 2003; Bethancourt et al., 2011; Daviu et al., 2014; Day, Reed, & Stevenson, 2016), raising the possibility that the reduction in contextual fear shown by females simply reflects that they are more active. However, several studies have reported no sex differences in cued fear conditioning (Baker‐Andresen, Flavell, Li, & Bredy, 2013; Baran, Armstrong, Niren, Hanna, & Conrad, 2009; Chen et al., 2014; Clark et al., 2019; Fenton, Halliday, Mason, Bredy, & Stevenson, 2016; Fenton et al., 2014; Maes, 2002; Maren et al., 1994; Markus & Zecevic, 1997). This suggests that greater locomotion in females is unlikely to account for sex differences in contextual fear, although some studies have also found reduced cued fear conditioning in females (Baran, Armstrong, Niren, & Conrad, 2010; Clark et al., 2019; Kosten, Miserendino, Bombace, Lee, & Kim, 2005; Pryce, Lehmann, & Feldon, 1999). However, this reduction in contextual fear conditioning in females agrees with other evidence indicating reduced spatial‐related learning and memory performance more generally in comparison with males (Yagi & Galea, 2019).

Studies have also examined the involvement of gonadal hormones in mediating these sex differences in Pavlovian fear learning and memory encoding. Ovariectomy was shown to enhance contextual fear conditioning in females, such that it abolished sex differences in contextual fear, and estrogen replacement in ovariectomized females reversed this effect (Gupta et al., 2001; Matsuda et al., 2015). Gonadectomy has been found to reduce contextual but not cued fear conditioning in males (McDermott, Liu, & Schrader, 2012). Other studies have found that cued fear expression did not differ across the estrous cycle or after ovariectomy in females, while gonadectomy enhanced cued fear expression in males, and this effect was mitigated by testosterone in an androgen receptor‐dependent manner (Chen et al., 2014; Milad, Igoe, Lebron‐Milad, & Novales, 2009). Moreover, androgen receptor overexpression was found to reduce contextual fear conditioning, which was reversed by gonadectomy or androgen receptor antagonism (Ramzan, Azam, Monks, & Zovkic, 2018). These different roles for gonadal hormones in regulating contextual and cued fear may involve differences in their sites of action in the neural circuitry involved, which is discussed below. Studies on sex differences in fear conditioning in humans are also covered below.

## 4 SEX DIFFERENCES IN FEAR EXTINCTION

Fear extinction is the reduction in learned fear responding that occurs with repeated presentations of the conditioned cue or prolonged re‐exposure to the conditioned context in the absence of the US. There has been intense interest in delineating the psychological and neurobiological mechanisms underlying this type of learned fear inhibition, given that it forms the theoretical basis for exposure therapy and is deficient in anxiety‐related disorders such as PTSD (Sevenster et al., 2018; Singewald & Holmes, 2019; Tovote et al., 2015; Zuj et al., 2016). Recent studies have shown sex differences in the extinction of Pavlovian fear memory.

### 4.1 Rodent studies

In studies investigating cued fear extinction, females have been shown to express more learned fear during extinction training and/or subsequent extinction memory testing, suggesting that females show resistance to extinction (Baker‐Andresen et al., 2013; Baran et al., 2009; Clark et al., 2019; Fenton et al., 2014, 2016; Greiner, Müller, Norris, Ng, & Sangha, 2019; Petrovich & Lougee, 2011). In contrast, there has been less research conducted on sex differences in contextual fear extinction and the findings have been mixed. One study found reduced extinction of contextual fear in females, compared to males (Matsuda et al., 2015). Another study reported enhanced contextual fear extinction in females, although the interpretation of this finding is complicated by the decrease in contextual fear shown by females at the start of extinction training (Daviu et al., 2014). Females have been shown to exhibit more spontaneous fear recovery over time after cued or contextual fear extinction, compared to males (Fenton et al., 2014, 2016; Matsuda et al., 2015). Contextual fear expression during cued fear extinction was also found to be enhanced in females relative to males (Baran et al., 2009; Fenton et al., 2014, 2016), raising the possibility that there are sex differences in the contextual regulation of fear extinction. However, another study found no sex differences in fear renewal, which is the return of fear after extinction when the cue is presented in a different context (Maes, 2002). Other studies have found sex differences in fear extinction but only in combination with certain genetic or environmental factors. Ter Horst, Carobrez, Mark, Kloet, and Oitzl (2012) showed that forebrain‐specific deletion of the mineralocorticoid receptor gene reduced fear extinction in females but not in males. Exercise has been shown to improve extinction memory and reduce later fear renewal in males but not females (Bouch et al., 2017).

**Figure 2:**
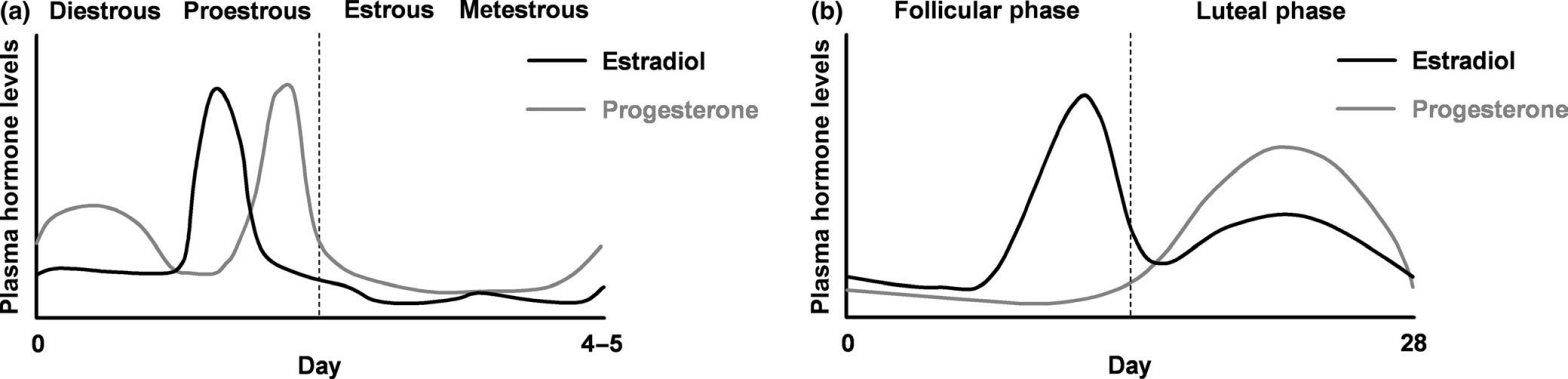
Schematic representation of fluctuations in the levels of estrogen and progesterone over the course of the (a) estrous cycle in female rats and (b) menstrual cycle in women. Vertical dotted lines represent the approximate time of ovulation (adapted from Maeng & Milad, 2015)

Whether females show reduced fear extinction in relation to males can be influenced by gonadal hormones. Milad et al. (2009) found no sex differences in cued extinction recall unless variations in estrous cycle phase were accounted for in the females. Extinction during metestrus, when estrogen and progesterone levels are lower, resulted in reduced extinction recall in comparison with males and to females extinguished during proestrus, when estrogen and progesterone levels are higher (Figure 2a). Other studies have found enhanced extinction recall when extinction was conducted during proestrus, compared to other estrous cycle phases (Gruene, Roberts, Thomas, Ronzio, & Shansky, 2015a; Rey, Lipps, & Shansky, 2014). Reduced cued extinction learning has been shown during diestrus, when gonadal hormone levels are also lower, compared to proestrus (Blume et al., 2017). Moreover, estradiol or progesterone given to females undergoing cued extinction during metestrus resulted in enhanced extinction recall, whereas blocking estrogen or progesterone signaling in females extinguished during proestrus reduced extinction recall (Maeng, Cover, et al., 2017a; Milad et al., 2009). Estrogen receptor beta (ERβ), but not estrogen receptor alpha (ERα), agonist treatment before cued fear extinction during metestrus was found to enhance extinction recall, while estradiol given immediately, but not 4 hr, after extinction also enhanced later extinction recall (Zeidan et al., 2011). Ovariectomy or hormonal contraceptive treatment, both of which reduce circulating gonadal hormone levels, has been shown to reduce cued fear extinction in females (Graham & Milad, 2013; Parrish, Bertholomey, Pang, Speth, & Torregrossa, 2019). This effect of hormonal contraceptives can be reversed by ERα or ERβ agonist treatment (Graham & Milad, 2013). Another study found that estradiol given during cued fear extinction enhanced subsequent extinction recall, whereas progesterone had different effects depending on when it was administered relative to extinction. Progesterone facilitated the effect of estradiol on extinction recall when given 6 hr before extinction training but blocked the facilitatory effect of estradiol on extinction recall when given 24 hr before extinction training (Graham & Daher, 2016). Estrogen regulation of cued fear extinction was found to be dose‐dependent and modulated by NMDA receptor signaling (Graham & Scott, 2018a, 2018b). Both testosterone and estrogen can also regulate cued fear extinction in males. Gonadotropin‐releasing hormone agonist treatment before extinction increased circulating testosterone levels and enhanced extinction recall in one study (Maeng, Taha, et al., 2017b), while another study showed that inhibiting estradiol synthesis during extinction impaired later extinction recall in males (Graham & Milad, 2014). These findings indicate that estrogen and testosterone enhance the encoding of cued extinction memory, while progesterone may regulate extinction in a more complex manner.

The role of gonadal hormones in regulating contextual fear extinction is less clear. One study in naturally cycling females showed reduced extinction learning during proestrus, compared to diestrus (Blume et al., 2017). Another study showed that females conditioned during proestrus and estrus displayed enhanced extinction, compared to males and to females conditioned during diestrus. Estrogen, but not progesterone, also enhanced contextual fear extinction in ovariectomized females. Moreover, ERβ, but not ERα, agonism enhanced contextual fear extinction in ovariectomized females (Chang et al., 2009). However, McDermott, Liu, Ade, and Schrader (2015) showed that chronic treatment with high levels of estradiol reduced contextual fear extinction. These discrepancies might involve methodological differences between the studies (e.g., estrous cycle phase during conditioning vs. extinction, naturally cycling vs. ovariectomized females, acute vs. chronic drug treatment). The potential roles of progesterone and testosterone in modulating contextual fear extinction remain unclear.

Sex differences in the return of learned fear after extinction have also been shown in juveniles, where fear renewal, reinstatement (the return of fear after non‐reinforced US exposure) and spontaneous fear recovery were observed in prepubescent females but not males (Park, Ganella, & Kim, 2017). This suggests that such sex differences in fear relapse after extinction can occur independently of the postpubertal activational effects of gonadal hormones and may instead involve their organizational effects during early development (Donner & Lowry, 2013). Alternatively, these sex differences may occur independently of gonadal hormone signaling and involve genetic and epigenetic effects, or possibly interactions between these different factors (Ratnu, Emami, & Bredy, 2017).

### 4.2 Human studies

Compared to the rodent literature, there have been far fewer comparable human studies conducted in healthy volunteers that have investigated this issue and demonstrated sex differences in fear extinction, unless variations in the gonadal hormone status of the women were taken into account (see below). One of the first such studies to be conducted found no sex differences in cued fear extinction (Fredrikson, Hugdahl, & Ohman, 1976). A recent study used a cued fear conditioning paradigm with a highly aversive film clip as the US to investigate sex differences in re‐experiencing intrusive memories related to the film clip, which were later elicited by the cue or occurred spontaneously after conditioning (Rattel et al., 2019). Cue‐triggered intrusive memories in this paradigm are thought to model conditioned responding to trauma reminders, which is a feature of PTSD, and may thus resemble real‐life traumatic experiences more than the aversive stimuli that are typically used in such studies (Wegerer, Blechert, Kerschbaum, & Wilhelm, 2013). Women were found to experience more of these intrusive memories and associated distress, compared to men. Moreover, this sex difference in intrusive memories was linked to reduced fear extinction in women, compared to men (Rattel et al., 2019).

As has been reported in rodent studies, sex differences in fear extinction in healthy volunteers emerge when women are stratified according to their gonadal hormone status. Milad et al. (2006) investigated cued fear conditioning and extinction in men and in women in the early or late follicular phases of the menstrual cycle, when estrogen is at lower or higher levels, respectively (Figure 2b). Men showed more fear conditioning than women, who did not differ based on their menstrual cycle phase. During extinction, there were no sex or menstrual cycle phase differences. However, women in the late follicular phase showed reduced extinction recall, compared to men and to women in the early follicular phase, suggesting an inverse relationship between estrogen levels and extinction memory. Another study replicated this sex difference in fear conditioning but found instead that higher levels of estradiol during extinction learning were associated with enhanced extinction recall in women. Furthermore, women with low levels of estradiol during extinction showed reduced extinction recall, compared to men and to women with high levels of estradiol (Milad et al., 2010). This relationship between estradiol levels during extinction and fear during later extinction recall in healthy women has been replicated in subsequent studies (Li & Graham, 2016; White & Graham, 2016; Zeidan et al., 2011). Lower levels of estradiol have also been associated with higher fear during extinction and intrusive memory strength in healthy women (Wegerer, Kerschbaum, Blechert, & Wilhelm, 2014). The contradictory findings of Milad et al. (2006) in comparison with these more recent studies might have resulted from relating extinction recall to the phase of the estrous cycle instead of directly to gonadal hormone levels. It is possible that variability in cycle length, hormone levels or accuracy of cycle phase determination may contribute to such discrepancies (Graham, Li, Black, & Öst, 2018; Pineles et al., 2016). The potential involvement of progesterone and testosterone in contributing to sex differences in fear extinction in humans is still incompletely understood (Milad et al., 2010; Pace‐Schott et al., 2013; Wegerer et al., 2014; Zeidan et al., 2011).

There is evidence for sex differences in fear conditioning and extinction in PTSD. Women with PTSD were found to show enhanced fear conditioning, compared to men with PTSD (Inslicht et al., 2013). Shvil et al. (2014) found that men with PTSD had reduced extinction recall, compared to women with PTSD, whereas there were no sex differences in fear conditioning or extinction in healthy trauma‐exposed controls. Gonadal hormone status can also influence fear extinction in anxiety‐related disorders. One study investigated associations between estradiol levels and fear conditioning and extinction in women with or without PTSD (Glover et al., 2012). There was no link between estradiol and fear conditioning in either group. However, low estradiol levels were associated with higher fear expression during extinction but only in women with PTSD. Another study found that women with PTSD showed reduced extinction recall, compared to women without PTSD. Moreover, while low levels of estradiol and progesterone predicted reduced extinction recall in women without PTSD, reduced extinction recall in women with PTSD was predicted by low estradiol and high progesterone levels (Pineles et al., 2016). Li and Graham (2016) found that, compared to high estradiol levels, low levels of estradiol during extinction in women with spider phobia resulted in greater fear during later extinction recall. Similarly, in spider phobic women undergoing a single session of exposure therapy, hormonal contraceptive use and lower estradiol levels during the session were associated with poorer outcomes post‐treatment (Graham et al., 2018). Taken together, these findings suggest that the relationship between estrogen and extinction is similar in women with or without PTSD, while the role of progesterone is, again, less clear.

## 5 SEX DIFFERENCES IN FEAR DISCRIMINATION

In addition to aberrant fear memory persistence and impaired fear extinction, certain anxiety‐related disorders are characterized by the overgeneralization of fear in response to harmless cues or contexts. This feature of such disorders has been investigated by examining fear discrimination and generalization using analogous behavioral paradigms across animal and human studies. In these paradigms, one cue or context (CS+) typically becomes associated with the US, while another cue or context (CS−) does not, resulting in the CS+ and CS‐ predicting threat and safety, respectively. Similarly, in conditioned inhibition (e.g., A+/AB−) paradigms, one cue typically becomes associated with the US to predict threat (A+) unless it is presented in conjunction with another cue (B), in which case presentation of the two cues together results in no US presentation (AB−) and instead signals safety (Christianson et al., 2012). Fear discrimination is thought to be a form of learned fear inhibition by the safety signal. Moreover, fear generalization in anxiety‐related disorders has been conceptualized as a deficit in fear inhibition resulting from deficient safety signaling and may represent a biomarker for these disorders (Asok, Kandel, et al., 2019a; Dunsmoor & Paz, 2015; Jovanovic et al., 2012). Emerging evidence indicates that there are sex differences in fear discrimination and safety signaling.

### 5.1 Rodent studies

Studies have shown less contextual fear discrimination (i.e., more generalization) in females, compared to males (Asok, Hijazi, et al., 2019b; Keiser et al., 2017; Lynch, Cullen, Jasnow, & Riccio, 2013; Reppucci, Kuthyar, & Petrovich, 2013). One study also reported reduced contextual fear discrimination in females, compared to males, in response to predator odor stress (Homiack, O'Cinneide, Hajmurad, Dohanich, & Schrader, 2018). This sex difference in contextual fear discrimination has been shown to depend on the time between conditioning and retrieval memory testing, such that generalization in females only emerged over several days after conditioning (Lynch et al., 2013). However, other studies have found no sex differences in contextual fear discrimination or in the incubation of generalized fear over time (Germer, Kahl, & Fendt, 2019; Keeley, Bye, Trow, & McDonald, 2015). Methodological differences (e.g., conditioned responses used to index fear) between the studies may help to explain these discrepancies. It is also worth noting that studies on sex differences in contextual fear discrimination using naturally cycling females have not systematically investigated the potential contributions of estrous cycle phase or gonadal hormone levels (e.g., compared to the fear extinction literature), which may provide another explanation for these discrepant findings.

Estrogen can influence contextual fear discrimination, but, in contrast to its facilitatory effect on fear extinction, estrogen has been found to reduce fear discrimination in females. Ovariectomy was shown to prevent the emergence of contextual fear generalization over time, whereas ovariectomy combined with estradiol replacement resulted in similar generalized fear over time as in naturally cycling females (Lynch et al., 2013). This effect of estradiol was found to be mediated by regulating memory retrieval in an ERβ‐dependent manner (Lynch et al., 2014). Interestingly, testosterone has been shown to reduce contextual fear generalization in males through its aromatization to estrogen (Lynch, Vanderhoof, et al., 2016a). Gonadectomized males were found to generalize their fear, which was prevented by testosterone or estradiol treatment; the latter effect involved both ERα and ERβ signaling. However, gonadectomized males treated with a non‐aromatizable androgen or an aromatase inhibitor showed fear generalization. Taken together, these findings suggest that estrogen has opposing effects on contextual fear discrimination in females and males, with estrogen reducing discrimination in females and enhancing discrimination in males. A role for progesterone in modulating contextual fear discrimination has yet to be established.

More recent studies have investigated sex differences in cued fear discrimination, but their findings have also been mixed. Males and females have been shown to discriminate and generalize, respectively, between cues predicting threat or safety (Aranda‐Fernandez, Gaztañaga, Arias, & Chotro, 2016; Day et al., 2016; Greiner et al., 2019). However, other studies have shown successful cued fear discrimination in both males and females (Clark et al., 2019; Gilman, DaMert, Meduri, & Jasnow, 2015), or even greater fear discrimination in females, compared to males (Foilb, Bals, Sarlitto, & Christianson, 2017). Another study showed that adversity in adolescence resulted in a later reduction in discrimination between cues predicting threat, uncertainty and safety in females but not males, although the female controls did successfully distinguish between these different cues (Walker, Andreansky, Ray, & McDannald, 2018). Day et al. (2016) found that the nature of sex differences in fear discrimination during retrieval testing depended on the extent of training received. Males showed successful discrimination after extended, but not limited training, whereas females discriminated after limited training but generalized after extended training. Discrimination in males involved safety signaling by the CS−, and generalization in females was due to reduced safety signaling by the CS− as shown by a retardation test, where the CS− was used as the cue during subsequent fear conditioning (Christianson et al., 2012). If the CS− signaled safety during discrimination, then later conditioning to the CS‐ was expected to be reduced (or retarded), which was the case for males but not females (Day et al., 2016). A summation test can also demonstrate safety signaling by the CS− during fear discrimination, where presenting the CS+ and CS− together would be expected to reduce fear in comparison with CS+ presentation alone. In conditioned inhibition paradigms, summation testing can demonstrate the inhibitory properties of B, where presenting B together with A+ or another conditioned cue would be expected to reduce conditioned responding relative to presentation of either conditioned cue in the absence of B (Christianson et al., 2012). Some studies have shown conditioned inhibition in males but not females (Aranda‐Fernandez et al., 2016; Greiner et al., 2019), while another study found no sex differences in conditioned inhibition (Foilb et al., 2017). However, estrous cycle phase was not determined in the studies that used naturally cycling females to examine sex differences in cued fear discrimination or conditioned inhibition. Again, this raises the possibility that variations in gonadal hormone levels between studies may account for these contradictory findings.

In contrast to contextual fear discrimination, little research has investigated gonadal hormone regulation of cued fear discrimination and safety signaling. Toufexis, Myers, Bowser, and Davis (2007) found that gonadectomized males and females both showed conditioned inhibition, which was disrupted by estradiol replacement but only in females. This suggests a sex‐specific role for estrogen in regulating safety signaling. Whether progesterone or testosterone play roles in sex differences in cued fear discrimination or safety signaling remains unknown. A sex difference in conditioned inhibition has also been shown in juveniles, where prepubescent males but not females showed conditioned inhibition (Aranda‐Fernandez et al., 2016). This suggests that such sex differences may also involve the organizational effects of gonadal hormones, genetic and epigenetic effects, or interactions between some or all of these factors.

### 5.2 Human studies

Compared to fear extinction, less research has been conducted on sex differences in fear discrimination in humans. In one study in healthy volunteers, women showed reduced contextual fear discrimination, compared to men. Moreover, women taking hormonal contraceptives discriminated less than free‐cycling women, although there were no differences in discrimination when comparing between the follicular and luteal phases of the menstrual cycle (Lonsdorf et al., 2015). Glover et al. (2013) found that cued fear discrimination and safety signaling were reduced with low, compared to high, estradiol levels in both non‐traumatized and traumatized women. Similarly, safety signaling was shown to be reduced with low, compared to high, estradiol levels in women with or without spider phobia (Li & Graham, 2016). However, a study investigating cued fear discrimination during pregnancy, when gonadal hormone levels are high, found that while non‐pregnant women showed discrimination, pregnant women showed generalization that was associated with PTSD symptoms (Michopoulous et al., 2015). It is worth noting that the hormonal milieu during pregnancy is very different from the menstrual cycle, suggesting a possible explanation for this discrepant finding. Another study in traumatized children found reduced cued fear discrimination in girls, compared to boys (Gamwell et al., 2015), suggesting that such sex differences may also occur independently of any activational effects of gonadal hormones.

## 6 NEURAL MECHANISMS IMPLICATED IN SEX DIFFERENCES IN LEARNED FEAR AND ITS INHIBITION

The neural circuitry underlying different Pavlovian fear learning and memory processes is distributed across various interconnected brain areas, including the amygdala, hippocampus and PFC. During contextual fear conditioning, the hippocampus and basolateral amygdala (BLA) are important for encoding the contextual representation and context–US association, respectively. In contrast, the lateral amygdala (La) is vital for associating the CS and US during cued fear conditioning, while the central amygdala (CeA) mediates learned fear expression. The medial PFC (mPFC) plays a key role in both contextual and cued fear memory processing. This neural circuit is also important for learned fear inhibition through extinction and discrimination (Anagnostaras, Gale, & Fanselow, 2001; Asok, Kandel, et al., 2019a; Dunsmoor & Paz, 2015; Sevenster et al., 2018; Tovote et al., 2015). Evidence indicates that sex differences in fear conditioning, extinction and discrimination involve each of these brain areas.

### 6.1 Rodent studies

Reduced contextual fear conditioning in females, compared to males, was found to be associated with diminished long‐term potentiation (LTP), a model of learning‐induced synaptic plasticity, in the dorsal hippocampus (DH) (Maren et al., 1994). This sex difference in contextual fear conditioning has also been related to decreased levels of phosphorylated extracellular signal‐regulated kinase (pERK), an important molecular mediator of memory encoding, in the ventral hippocampus in females (Gresack et al., 2009). In terms of the inhibitory effect of estrogen on contextual fear conditioning, Gupta et al. (2001) found that estradiol treatment after ovariectomy decreased LTP in DH, compared to ovariectomized controls treated with vehicle. Ovariectomy has also been shown to reduce LTP in La, which was reversed by estradiol. The enhancement of cued fear conditioning in males resulting from gonadectomy was found to be associated with enhanced LTP in La, and this was reversed by testosterone (Chen et al., 2014). Reduced contextual fear conditioning caused by androgen receptor overexpression in males was shown to be related to alterations in the expression of various plasticity‐related genes in DH (Ramzan et al., 2018). Enhanced learned fear expression in females, compared to males, was found to be associated with greater neuronal activity and excitability in both La and BLA (Blume et al., 2017). In a study examining the brain mechanisms involved in the cessation of feeding by learned fear cues, it was shown that different neural circuits were activated by feeding and fear, except that mPFC was recruited under both conditions in females but not males (Reppucci & Petrovich, 2018). Another study investigating the effects of mPFC lesions on cued fear learning found that such lesions enhanced the rate of conditioning in females but not males (Baran et al., 2010). Compared to males, PAC1 receptor expression in mPFC was found to be higher in females and blocking PAC1 receptor signaling in mPFC reduced trace fear conditioning, a paradigm in which a brief interval occurs between CS and US presentation, in females but not males (Kirry et al., 2018).

Sex differences in fear extinction have been linked to mPFC function. Lesions to mPFC have been shown to reduce cued fear extinction learning in females but not males, whereas mPFC lesions reduced the later recall of extinction in males but not females (Baran et al., 2010). Extinction encoding is regulated by brain‐derived neurotrophic factor (BDNF), an important mediator of synaptic plasticity, acting in mPFC in males (Peters, Dieppa‐Perea, Melendez, & Quirk, 2010; Rosas‐Vidal, Do‐Monte, Sotres‐Bayon, & Quirk, 2014). Resistance to cued fear extinction in females, compared to males, was found to be related to reduced BDNF expression in mPFC involving increased methylation of the BDNF gene in this area. Furthermore, agonism of the tropomyosin‐related kinase B (TrkB) receptor, which mediates BDNF signaling, in mPFC reduced cued fear expression during extinction and later fear renewal in females (Baker‐Andresen et al., 2013). Studies in males have shown that the prelimbic (PL) and infralimbic (IL) subregions of mPFC mediate learned fear expression and extinction, respectively (Sierra‐Mercado, Padilla‐Coreano, & Quirk, 2011; Vidal‐Gonzalez, Vidal‐Gonzalez, Rauch, & Quirk, 2006). Enhanced cued fear expression and spontaneous fear recovery in females, compared to males, were found to be associated with persistent theta and gamma oscillatory activity in PL and a failure of gamma activation in IL (Fenton et al., 2014, 2016). In a study investigating the neural correlates of individual differences in fear extinction, differences in the morphology of IL neurons receiving projections from BLA were found between subpopulations of males displaying high or low levels of extinction recall. However, there were no morphological differences in these neurons between females who displayed high or low extinction recall, suggesting that individual differences in extinction recall were mediated by distinct neural processes in females and males (Gruene, Roberts, et al., 2015a). Another study found that reduced contextual fear extinction in females, compared to males, was associated with decreased pERK expression in DH but not mPFC (Matsuda et al., 2015).

Gonadal hormone regulation of fear extinction also involves this neural circuitry. A study found differences in cued and contextual fear extinction during diestrus and proestrus that were associated with differences in neuronal excitability in La and BLA during these estrous cycle phases. Cued fear extinction and La inhibition were enhanced during proestrus, compared to diestrus, while contextual fear extinction and BLA inhibition were enhanced during diestrus, compared to proestrus (Blume et al., 2017). Compared to vehicle, females treated with estradiol after cued fear extinction were shown to have lower amygdala and higher IL activation during later extinction recall (Zeidan et al., 2011). Another study found that females given estradiol before cued fear extinction showed enhanced extinction recall and a corresponding decrease in CeA activation and an increase in the ratio of activation between IL‐PL and IL‐CeA (Maeng, Taha, et al., 2017b). A study investigating dopamine–estrogen interactions underlying cued fear extinction in females found opposing effects of D1 receptor agonism during proestrus versus other estrous cycle phases on later extinction recall and accompanying activation of IL neurons receiving BLA projections. Rey et al. (2014) found that D1 receptor agonist treatment before extinction during proestrus impaired extinction recall and this was associated with reduced IL activation. Conversely, D1 receptor agonism before extinction during other phases of the estrous cycle enhanced extinction recall, which was related to augmented IL activation. Estradiol has been shown to potentiate synaptic plasticity in an ERβ‐ and NMDA receptor‐dependent manner in IL (Galvin & Ninan, 2014), suggesting a mechanism by which estrogen enhances extinction memory encoding. Estradiol or ERβ agonism has been shown to enhance contextual fear extinction in ovariectomized females by acting in DH (Chang et al., 2009). In contrast, reduced contextual fear extinction resulting from chronic treatment with high levels of estradiol has been associated with reduced COMT levels in DH but not mPFC (McDermott et al., 2015).

Compared to fear conditioning and its extinction, few studies have examined the neural mechanisms involved in sex differences in fear discrimination. One study showed that contextual fear discrimination in males was associated with DH activation, whereas reduced discrimination in females was linked more to BLA recruitment (Keiser et al., 2017). Another study showed that reduced contextual fear discrimination with predator odor stress in females, compared to males, was associated with sex differences in signaling by cAMP response element binding protein, a crucial molecular mediator of learning and memory, in DH (Homiack et al., 2018). Contextual fear generalization induced by estradiol treatment was found to depend on ERβ activation in DH (Lynch, Winiecki, Vanderhoof, Riccio, & Jasnow, 2016b). More recently, the anterior cingulate cortex (ACC) was found to be a site of action for the effects of estradiol on contextual fear generalization. Glutamate receptor signaling in DH and ACC was also recently shown to be involved in mediating this effect of estradiol, given that AMPA or NMDA receptor antagonism in these areas blocked contextual fear generalization induced by estradiol (Adkins, Lynch, Hagerdorn, Esterhuizen, & Jasnow, 2019). In contrast to contextual fear discrimination, the neural underpinnings of sex differences in cued fear discrimination and safety signaling remain poorly understood.

### 6.2 Human studies

Recent studies investigating brain function in very large cohorts of healthy volunteers have shown clear sex differences in the resting‐state connectivity of neural circuits comprising the hippocampus, amygdala and mPFC (Conrin et al., 2018; Zhan et al., 2017). Evidence also indicates that there are sex differences in the activity of these areas in relation to fear conditioning and extinction. One study found that, compared to men, women showed more activation of the amygdala and ACC during cued fear conditioning. No sex differences in amygdala or ACC activation were found during extinction. However, during extinction recall, men showed higher activity in ACC, compared to women (Lebron‐Milad et al., 2012). Gonadal hormone status modulates brain activation in relation to fear extinction in healthy women. Higher estradiol levels during extinction have been associated with increased activation of ventromedial PFC (vmPFC), which is thought to be the human homolog of the rodent IL. These higher estradiol levels during extinction were also found to be related to enhanced extinction recall concomitant with increased activation of vmPFC and amygdala (Zeidan et al., 2011). Another study found that women taking oral contraceptives showed more activation in amygdala, ACC and vmPFC during cued fear extinction, compared to women in the luteal phase of the menstrual cycle (Merz et al., 2012). The brain areas involved in sex differences in fear discrimination in humans remain to be elucidated.

Genetic factors that have been identified to moderate risk for anxiety‐related disorders depending on sex can influence amygdala and hippocampus function. COMT genotype was found to regulate amygdala activation in response to threat‐related stimuli in healthy women but not men (Domschke et al., 2012). PAC1 receptor genotype has been shown to modulate activity in the hippocampus during contextual fear conditioning in healthy women (Pohlack et al., 2015). PAC1 receptor genotype in traumatized women was also found to regulate activity in and connectivity between the amygdala and hippocampus in response to threatening stimuli (Stevens et al., 2014). Studies have shown sex differences in PFC function in anxiety‐related disorders. In dental phobia, men were found to have greater PFC activation in response to fear‐related stimuli, compared to women (Schienle, Köchel, & Leutgeb, 2011; Schienle, Scharmüller, Leutgeb, Schäfer, & Stark, 2013). In a study investigating brain function in relation to cued fear extinction in PTSD, no sex differences were found in healthy trauma‐exposed controls, but men with PTSD showed reduced extinction recall linked to enhanced ACC activation, compared to women with PTSD (Shvil et al., 2014).

## 7 FUTURE DIRECTIONS

The evidence reviewed above indicates that there are important sex differences in learned fear and its inhibition, which are influenced by gonadal hormone signaling and accompanied by differences in the function of the relevant neural circuitry (summarized in Tables 1, 2, 3, 4). On balance, studies across species have shown that both fear extinction and discrimination are reduced in females, compared to males. Rodent and human studies are also in general agreement with respect to estrogen regulation of fear extinction in females. However, less research has investigated the role of gonadal hormones in regulating fear discrimination and the findings from the rodent and human studies conducted to date seem contradictory. Given that impaired inhibition of learned fear and associated neural circuit dysfunction are characteristic of certain anxiety‐related disorders, further investigation of the neurobiological basis of sex differences in learned fear inhibition is a potentially fruitful area of research to better understand why women are so much more prone to developing these disorders than men. Below we suggest various lines of enquiry for future work in this area.

**Table 1:** Summary of studies on sex differences in and gonadal hormone regulation of fear extinction in rodents

Subjects	Measures/manipulations	Differences/effects	Neural mechanism(s)	Reference
M and F rats	Cued extinction learning	*M* > F	Not examined	Baran et al. (2009)
M and F rats	mPFC lesion effects on cued extinction learning and recall	Lesions ↓ extinction learning in F and ↓ recall in M	mPFC lesions	Baran et al. (2010)
M and F rats	Cued extinction learning	*M* > F	Not examined	Petrovich and Lougee (2011)
M and F mice	Cued extinction recall	*M* > F	BDNF DNA methylation and mRNA expression in mPFC	Baker‐Andresen et al. (2013)
F mice	TrkB‐R agonist effects on cued extinction and fear renewal	↓ cued fear expression during extinction and ↓ fear renewal	TrkB‐R signaling in mPFC	Baker‐Andresen et al. (2013)
M and F rats	Cued fear expression Spontaneous fear recovery	*F* > M *F* > M	PL theta activity PL theta activity	Fenton et al. (2014)
M and F rats	Cued fear expression Spontaneous fear recovery	*F* > M *F* > M	PL gamma activity IL gamma activity	Fenton et al. (2016)
M and F rats	Cued extinction recall	*M* > F	Not examined	Voulo & Parsons (2017)
M and F mice	Cued extinction learning	*M* > F	Not examined	Clark et al. (2019)
M and F rats	Cued extinction learning and recall	*M* > F	Not examined	Greiner et al. (2019)
M and F rats	Fear renewal after cued extinction	*M* = F	Not examined	Maes (2002)
M and F mice	Cued extinction learning	*M* > F	Forebrain‐specific deletion of MR gene	Ter Horst et al. (2012)
M and F rats	Exercise effects on cued extinction recall and fear renewal	↑ recall and ↓ fear renewal in M but not F	Not examined	Bouchet et al. (2017)
M and F rats	Cued extinction, during different estrous cycle phases in F	Extinction during Met ↓ recall versus extinction during Pro and M	Not examined	Milad et al. (2009)
F rats	Cued extinction during different estrous cycle phases	Extinction during Est/Met/Di ↓ recall versus extinction during Pro	Not examined	Rey et al. (2014)
F rats	D1‐R agonist effects on cued extinction during different estrous cycle phases	Cycle phase‐dependent effects on recall	IL activation	Rey et al. (2014)
F rats	Cued extinction during different estrous cycle phases	Extinction during Met/Di/Est ↓ extinction learning and recall versus extinction during Pro	Not examined	Gruene, Roberts, et al. (2015a)
M and F rats	Individual differences in cued extinction	Differences in recall linked to differences in IL neuron morphology in M but not FExtinction during	IL neuron morphology	Gruene, Roberts, et al. (2015a)
F rats	Cued extinction during different estrous cycle phases	Extinction during Di ↓ extinction learning versus extinction during Pro	La activity	Blume et al. (2017)
F rats	E or P effects during cued extinction in Met	E or P ↑ recall	Not examined	Milad et al. (2009)
F rats	ER or PR blockade effects during cued extinction in Pro	ER or PR blockade ↓ recall	Not examined	Milad et al. (2009)
F rats	ERα or ERβ agonist effects during cued extinction in Met	ERβ but not ERα agonism ↑ recall	Not examined	Zeidan et al. (2011)
F rats	E effects after cued extinction in Met	E given immediately but not 4 hr after extinction ↑ recall	Amygdala and IL activation	Zeidan et al. (2011)
F rats	E effects during cued extinction in Met	E ↑ recall	CeA activation and ratios of IL‐PL and IL‐CeA activation	Maeng, Cover, et al. (2017a)
F rats	HC effects on cued extinction	HC ↓ extinction learning and recall via ERα and ERβ	Not examined	Graham and Milad (2013)
F rats	OVX or HC effects on cued extinction	OVX/HC ↓ extinction learning and recall via E	Not examined	Parrish et al. (2019)
OVX F rats	E or E + P effects on cued extinction	E ↑ recall, time‐dependent modulation of E effects by P	Not examined	Graham &Daher (2016)
F rats	E effects on cued extinction during different estrous cycle phases	Dose‐ and cycle phase‐dependent effects on recall	Not examined	Graham and Scott (2018a)
Naturally cycling and OVX F rats	NMDA‐R modulation of E effects on cued extinction	E effects on extinction NMDA‐R‐dependent	Not examined	Graham and Scott (2018b)
M rats	GnRH agonist effects during cued extinction	GnRH agonism ↑ recall via ↑ T	Not examined	Maeng, Taha, et al. (2017b)
M rats	Aromatase inhibitor effects during cued extinction	Aromatase inhibition ↓ recall via ↑ E	Not examined	Graham and Milad (2014)
F rats	Contextual extinction during different estrous cycle phases	Extinction during Pro ↓ extinction learning versus extinction during Di	BLA activity	Blume et al. (2017)
Juvenile M and F rats	Fear relapse after cued extinction	Renewal, reinstatement and spontaneous recovery shown in F but not M	Not examined	Park et al. (2017)
M and F rats	Contextual fear conditioning, during different estrous cycle phases in F, and extinction	F conditioned during Pro or Est ↑ extinction versus F conditioned during Di and M	Not examined	Chang et al. (2009)
OVX F rats	E or P effects on contextual extinction	E but not P enhanced extinction	Not examined	Chang et al. (2009)
OVX F rats	ERα or ERβ agonist effects on contextual extinction	ERβ but not ERα agonism ↑ extinction	Not examined	Chang et al. (2009)
OVX F mice	Chronic E effects on contextual extinction	High dose of chronic E ↓ extinction	COMT expression in DH	McDermott et al. (2015)
M and F mice	Contextual extinction learning Spontaneous fear recovery	*M* > F *F* > M	pERK expression in DH	Matsuda et al. (2015)

Abbreviations: BDNF, brain‐derived neurotrophic factor; BLA, basolateral amygdala; CeA, central amygdala; COMT, catechol‐O‐methyltransferase; D1‐R, dopamine D1 receptor; DH, dorsal hippocampus; Di, diestrus; E, estradiol; ER, estrogen receptor; ERα, estrogen receptor α; ERβ, estrogen receptor β; Est, estrus; F, female; GnRH, gonadotropin‐releasing hormone; HC, hormonal contraceptive; IL, infralimbic cortex; La, lateral amygdala; M, male; Met, metestrus; mPFC, medial prefrontal cortex; MR, mineralocorticoid receptor; NMDA‐R, NMDA receptor; OVX, ovariectomized; P, progesterone; pERK, phosphorylated extracellular signal‐regulated kinase; PL, prelimbic cortex; PR, progesterone receptor; Pro, proestrus; T, testosterone; TrkB‐R, tropomyosin‐related kinase B receptor.

**Table 2:** Summary of studies on sex differences in and gonadal hormone regulation of fear extinction in humans

Subjects	Measures/ manipulations	Differences/ effects	Neural mechanism(s)	References
M and W	Extinction	*M* = W	Not examined	Fredrikson et al. (1976)
M and W	Extinction	*M* > W	Not examined	Rattel et al. (2019)
M and W	Extinction, during different menstrual cycle phases in W	Extinction in LF ↓ recall versus extinction in EF and M	Not examined	Milad et al. (2006)
M and W	Extinction, in W with low or high E	Low E during extinction ↓ recall versus high E during extinction and M	Not examined	Milad et al. (2010)
W	Low or high E during extinction	Low E during extinction ↓ recall versus high E during extinction	vmPFC and amygdala activity	Zeidan et al. (2011)
W	Low or high E during extinction	Low E during extinction ↓ recall versus high E during extinction	Not examined	White and Graham (2016)
W	Low or high E during extinction	Low E during extinction ↓ recall versus high E during extinction	Not examined	Wegerer et al. (2014)
M and W with PTSD	Extinction recall	W > M	ACC activity	Shvil et al. (2014)
W with PTSD	Low or high E during extinction	Low E ↓ extinction learning versus high E	Not examined	Glover et al. (2012)
W with PTSD	Extinction	Low E and high P during extinction predicted ↓ recall	Not examined	Pineles et al. (2016)
W with phobia	Low or high E during extinction	Low E during extinction ↓ recall versus high E during extinction	Not examined	Li and Graham (2016)
W with phobia	Low or high E during exposure therapy	Low E during exposure therapy predicted ↓ post‐treatment outcomes	Not examined	Graham et al. (2018)

Abbreviations: ACC, anterior cingulate cortex; E, estradiol; EF, early follicular; LF, late follicular; M, men; PTSD, post‐traumatic stress disorder; vmPFC, ventromedial prefrontal cortex; W, women.

**Table 3:** Summary of studies on sex differences in and gonadal hormone regulation of fear discrimination in rodents

Subjects	Measures/ manipulations	Differences/ effects	Neural mechanism(s)	References
M and F rats	Context discrimination	*M* > F	Not examined	Reppucci et al. (2013)
M and F rats	Context discrimination	*M* > F	Not examined	Lynch et al. (2013)
OVX F rats	E effects on context discrimination	OVX ↑ and OVX + E ↓ discrimination	Not examined	Lynch et al. (2013)
M and F mice	Context discrimination	*M* > F	DH and BLA activation	Keiser et al. (2017)
M and F mice	Context discrimination	*M* > F	Not examined	Asok, Hijazi, et al. (2019b)
M and F rats	Context discrimination with predator odor	*M* > F	CREB signaling in DH	Homiack et al. (2018)
M and F rats	Context discrimination	*M* = F	Not examined	Keely et al. (2015)
M and F mice	Context discrimination	*M* = F	Not examined	Germer et al. (2019)
OVX F rats	E, ERα agonist or ERβ agonist effects on context discrimination	E ↓ discrimination retrieval ERβ but not ERα agonism ↓ retrieval	Not examined	Lynch et al. (2014)
OVX F rats	ER antagonist effects on context discrimination	Blocked effects of E or ERβ agonism on discrimination	DH	Lynch, Winiecki, et al. (2016b)
OVX F rats	Glu‐R modulation of E effects on context discrimination	E effects on discrimination Glu‐R‐dependent	ACC and DH	Adkins et al. (2019)
GDX M rats	T, E, ERα agonist or ERβ agonist effects on context discrimination	T or E ↑ discrimination ERα or ERβ agonism ↑ discrimination	Not examined	Lynch, Winiecki, et al. (2016b)
M and F rats	Cued discrimination	*F* > M with limited training *M* > F with extended training	Not examined	Day et al. (2016)
M and F rats	Cued discrimination	Early adversity ↓ discrimination in F but not M	Not examined	Walker et al. (2018)
M and F mice	Cued discrimination	*M* = F	Not examined	Gilman et al. (2015)
M and F mice	Cued discrimination	*M* = F	Not examined	Clark et al. (2019)
M and F rats	Cued discrimination Conditioned inhibition	*F* > M *F* = M	Not examined	Foilb et al. (2017)
Juvenile M and F rats	Conditioned inhibition	*M* > F	Not examined	Aranda‐Fernandez et al. (2016)
M and F rats	Conditioned inhibition	*M* > F	Not examined	Greiner et al. (2019)
GDX M and OVX F rats	E effects on conditioned inhibition	E ↓ conditioned inhibition in F but not M	Not examined	Toufexis et al. (2007)

Abbreviations: ACC, anterior cingulate cortex; BLA, basolateral amygdala; CREB, cAMP response element binding protein; DH, dorsal hippocampus; E, estradiol; ER, estrogen receptor; ERα, estrogen receptor α; ERβ, estrogen receptor β; F, female; GDX, gonadectomized; Glu‐R, glutamate receptor; M, male; OVX, ovariectomized; T, testosterone.

**Table 4:** Summary of studies on sex differences in and gonadal hormone regulation of fear discrimination in humans

Subjects	Measures/ manipulations	Differences/ effects	Neural mechanism(s)	References
M and W	Context discrimination	M and free‐cycling W > W on HC	Not examined	Lonsdorf et al. (2015)
Healthy and traumatized W	E effects on cued discrimination and conditioned inhibition	Low E ↓ discrimination and conditioned inhibition versus high E	Not examined	Glover et al. (2013)
Healthy and spider phobic W	E effects on cued discrimination	Low E ↓ discrimination versus high E	Not examined	Li and Graham (2016)
Non‐pregnant and pregnant W	Cued discrimination	Non‐pregnant > Pregnant	Not examined	Michopoulous et al. (2015)
Traumatized boys and girls	Cued discrimination	Boys > Girls	Not examined	Gamwell et al. (2015)

Abbreviations: E, estradiol; HC, hormonal contraceptive; M, men; W, women.

Compared to rodent studies, the evidence for sex differences in learned fear inhibition in humans is less clear without accounting for the gonadal hormone status of women. Genetic and environmental factors are more easily controlled for in rodent studies, which might contribute to this disparity. Future studies examining learned fear inhibition in men and women could take certain genetic or environmental factors into account to determine whether this results in more robust sex differences emerging for this anxiety‐related endophenotype. For example, it would be interesting to stratify women in such studies based on gene (e.g., COMT, PAC1 receptor) variants that moderate the function of relevant brain areas and confer risk for developing anxiety‐related disorders in a sex‐dependent manner.

Both rodent and human studies show that sex differences in fear extinction do emerge when accounting for variations in the estrogen status of females, such that estradiol enhances extinction. Similarly, human studies on fear discrimination broadly agree with these fear extinction studies, with estrogen enhancing discrimination in women. In contrast, estrogen reduces fear discrimination and safety signaling in ovariectomized rodents, although whether fluctuating estrogen levels in naturally cycling females can also affect fear discrimination and safety signaling remains to be examined in a systematic manner. Further studies should investigate this important issue given the potential implications for using estrogen‐based medicines (e.g., as adjuncts to exposure therapy) to treat anxiety‐related disorders in the future.

Rodent studies indicate a role for progesterone in regulating fear extinction, but whether this is also the case for humans is less clear, while progesterone regulation of fear discrimination remains unknown. Progesterone has anxiolytic effects through its metabolite allopregnanolone, a neurosteroid that potentiates inhibitory transmission by facilitating GABA‐A receptor signaling. Moreover, progesterone metabolism has been implicated in anxiety‐related disorders in both men and women (Pineles et al., 2018; Rasmusson et al., 2019; Schule, Nothdurfter, & Rupprecht, 2014). However, facilitating inhibitory transmission may also interfere with the efficacy of psychological treatments such as exposure therapy by impairing fear extinction (Singewald, Schmuckermair, Whittle, Holmes, & Ressler, 2015). Therefore, further research on the modulation of learned fear inhibition and associated neural circuit function by progesterone‐related signaling is warranted.

Studies investigating sex differences in fear reconsolidation, along with their potential endocrine and neural underpinnings, have only recently emerged (Franzen, Giachero, & Bertoglio, 2019; Meir Drexler, Merz, Hamacher‐Dang, & Wolf, 2016; da Silva, Takahashi, Bertoglio, Andreatini, & Stern, 2016). Learned fear retrieval can destabilize the memory engram, which requires its reconsolidation to maintain, strengthen or update fear memory. Disrupting reconsolidation to weaken fear memory is a potential therapeutic strategy for treating anxiety‐related disorders, but most of the relevant studies to date have been conducted in males. Various boundary conditions, including memory strength, are important for determining whether memories undergo reconsolidation after retrieval, such that strong fear memory can be more resistant to reconsolidation disruption (Lee, Nader, & Schiller, 2017). Future studies could thus examine whether there are sex differences in the mechanisms underlying fear reconsolidation.

It is worth noting that the conclusions drawn above on sex differences in learned fear and its inhibition in rodents are based largely on studies that were conducted using Pavlovian conditioning paradigms that inferred learned fear from freezing behavior. Studies conducted using other paradigms (e.g., eye‐blink conditioning, active avoidance) or behavioral measures of learned fear (e.g., fear‐potentiated startle) have found enhanced learning in females, compared to males, which involves the organizational and activational effects of gonadal hormones (Dalla & Shors, 2009). A recent study found sex differences in cued fear extinction that were response‐specific, such that extinction was reduced in females, compared to males, based on fear‐potentiated startle but not freezing (Voulo & Parsons, 2017). Interpreting the results of studies investigating sex differences in learned fear and extinction based solely on freezing can also be challenging because not only are females more active generally but they can also adopt more active fear responding, compared to males (Arakawa, 2019). Recent studies have found that some females exhibited active “darting” behavior in addition to freezing during cued fear conditioning and extinction, whereas males were much more likely to express freezing instead of darting (Colom‐Lapetina, Li, Pelegrina‐Perez, & Shansky, 2019; Gruene, Flick, Stefano, Shea, & Shansky, 2015b). Another study found that females showed fear generalization based on freezing but discriminated based on darting (Greiner et al., 2019); however, the levels of darting were very low overall and other studies have reported negligible darting in females during fear conditioning, extinction or discrimination (Blume et al., 2017; Foilb et al., 2017). Therefore, more research is needed to further characterize this predominantly sex‐specific conditioned fear response. Determining the mechanisms underpinning active fear responding and its inhibition is relevant to understanding avoidance, a symptom associated with poor long‐term outcomes in anxiety‐related disorders (Hendricks et al., 2016). A study showed that PTSD symptoms were associated with experimentally conditioned avoidance, which was more pronounced in women than in men (Sheynin et al., 2017). Rodent studies have shown that the extinction of passive (i.e., freezing) and active (i.e., avoidance) fear responding is mediated by partially overlapping neural circuits (Bravo‐River, Roman‐Ortiz, Brignoni‐Perez, Sotres‐Bayon, & Quirk, 2014; Martinez‐Rivera, Bravo‐Rivera, Velázquez‐Díaz, Montesinos‐Cartagena, & Quirk, 2019), although potential sex differences in the brain mechanisms involved remain to be determined.

While it is of real interest that females and males display more active and passive forms of learned fear responding, respectively, this also makes it more difficult to determine the nature of sex differences based on the expression of different behaviors between the sexes. Future studies in rodents examining sex differences in learned fear inhibition could also measure physiological responses (e.g., sympathetic activation as measured by changes in heart rate or blood pressure) elicited by conditioned cues or contexts alongside behavior. This is done routinely in such human studies (i.e., skin conductance response) and may complement the behavioral measures by allowing for directly comparing between males and females. Determining the neural basis of sex differences in the physiological responses associated with learned fear and its inhibition may also enhance the translational validity of rodent studies.

## 8 CONCLUSION

When considered from an adaptive perspective, resistance to extinction may promote learned fear responding in the face of cues or contexts that were once but are no longer predictive of threat. Similarly, generalizing across cues or contexts may promote learned fear in response to a wider range of more ambiguous stimuli or environments that can potentially predict threat. In evolutionary terms, such circumstances may actually confer a survival advantage to females and the offspring in their care. However, this phenotype related to risk aversion, which recent rodent studies have shown is enhanced in females relative to males (Orsini, Willis, Gilbert, Bizon, & Setlow, 2016; Pellman, Schuessler, Tellakat, & Kim, 2017), may also contribute to the greater vulnerability to anxiety‐related disorders that is seen in women in comparison with men.

## CONFLICT OF INTEREST

The authors have no competing interests to declare.

## AUTHOR CONTRIBUTIONS

HLLD and CWS drafted and revised the paper and approved the final version.

## Data Availability

Not applicable (this review reports no primary data).
